# Osteogenic Peptides and Attachment Methods Determine Tissue Regeneration in Modified Bone Graft Substitutes

**DOI:** 10.3390/jfb12020022

**Published:** 2021-03-31

**Authors:** George Bullock, Joss Atkinson, Piergiorgio Gentile, Paul Hatton, Cheryl Miller

**Affiliations:** 1 School of Clinical Dentistry, The University of Sheffield, Sheffield S10 2TA, UK; g.d.bullock@sheffield.ac.uk (G.B.); joss.atkinson@gmail.com (J.A.); c.a.miller@sheffield.ac.uk (C.M.); 2 School of Engineering, Newcastle University, Stephenson Building, Newcastle upon Tyne NE1 7RU, UK; piergiorgio.gentile@newcastle.ac.uk

**Keywords:** bone repair material, biomimetic peptides, surface functionalisation, tissue engineering

## Abstract

The inclusion of biofunctional molecules with synthetic bone graft substitutes has the potential to enhance tissue regeneration during treatment of traumatic bone injuries. The clinical use of growth factors has though been associated with complications, some serious. The use of smaller, active peptides has the potential to overcome these problems and provide a cost-effective, safe route for the manufacture of enhanced bone graft substitutes. This review considers the design of peptide-enhanced bone graft substitutes, and how peptide selection and attachment method determine clinical efficacy. It was determined that covalent attachment may reduce the known risks associated with growth factor-loaded bone graft substitutes, providing a predictable tissue response and greater clinical efficacy. Peptide choice was found to be critical, but even within recognised families of biologically active peptides, the configurations that appeared to most closely mimic the biological molecules involved in natural bone healing processes were most potent. It was concluded that rational, evidence-based design of peptide-enhanced bone graft substitutes offers a pathway to clinical maturity in this highly promising field.

## 1. Introduction

With an ageing population, there is a growing need for improved bone tissue regeneration [1,2,3]. After injury, given the appropriate conditions, bone tissue has the capacity to regenerate fully [4]. Healing, however, can be compromised by a number of factors including defect size, underlying disease conditions and/or poor vascularisation, resulting in a non-healing defect or fracture non-union which lacks the ability to complete self-repair processes. The prevalence of non-union can range from 2.5 to 46%, depending on the severity of the defect and age of the patient, and other local factors [5,6]. These clinical challenges are accompanied by a reduced quality of life and therefore surgical intervention is required to restore health and function. With the prevalence of non-union post therapy currently at 10%, there is a growing demand for enhanced osteoconductive and osteoinductive medical devices for bone tissue regeneration [7,8,9]. Osteoconduction is the process of bone cells and tissues regenerating at the surface of a scaffold biomaterial such as hydroxyapatite (HA), following implantation into established bony tissue; while osteoinduction is the biochemical stimulation of bone tissue regeneration through the recruitment and directed differentiation of mesenchymal stem cells (MSCs), and is associated with autografts and specific alloplastic grafts that have been modified with orthobiologic and related agents [4,10,11,12]. While advances in the development of osteoinductive bone grafts have improved the consistency of bone healing for many patients, they have also introduced new clinical complications, and challenges remain among elderly or compromised patients [13,14].

Although autografts are considered osteoinductive and the “gold standard”, the requirement for a second surgical site (with pain and a risk of infection or donor site morbidity) means this is not an ideal solution. The medical research community is therefore focused on further developing alloplastic graft substitutes through the incorporation of molecules to promote bone healing, rendering them osteoinductive, thereby alleviating the limitations and concerns surrounding other grafting materials [15]. The unmet clinical need for alloplastic bone graft substitutes, which provide consistent and predictable healing of bone defects led to the development and application of orthobiologics such as bone morphogenetic protein (BMP) 2 and 7, in the form of adsorbed protein inclusions [12,15,16,17,18].

Two BMPs are currently FDA approved for clinical use: recombinant human (rh) BMP-2 and rhBMP-7, with BMP-2 thought the most effective and included in a clinical product: Infuse^®^, produced by Medtronic Inc.©, is a collagen sponge carrier for rhBMP-2, for spinal fusion. However, the clinical outcomes related to the use of rhBMP-2 are disputed in the literature. Successful bone regeneration through the use of rhBMP-2 is reported in a plethora of multi-centre trails [19,20,21]. There is also a large amount of published work claiming no significant enhancement of non-union rates, and even adverse events associated with its use, many of which are summarised in a review written by Epstein in 2013 focused on complications in spine surgery related to Infuse^®^ [4,10,15,16,22]. These issues are believed to be the result of the high concentrations used, non-specific action and uncontrolled ectopic effects when the BMP has travelled in large concentrations to undesirable regions of the body. BMP-7 has also been incorporated into a medical product. OP-1, produced by Stryker^©^, contains rhBMP-7, and again, the published literature is in disagreement regarding its effectiveness. It has been shown that when used, in combination with a hydrogel carrier and bovine collagen type 1, rhBMP-7 stimulates the healing of non-unions at a rate similar to that of an autograft [23], while conversely, in another article similar healing patterns were conveyed in sites treated and untreated with rhBMP-7, indicating no significant differences [24]. The inability to predict or control tissue responses to orthobiologic grafts is associated with excessive bone tissue regeneration which is harmful to patients [4,10].

The use of smaller biomolecules, specifically small-chain peptides developed from certain domains within larger biomolecules, offers a potential solution to the issues surrounding larger biomolecule inclusions. Peptides are thought to allow for significantly greater control over cellular interactions in terms of consistency and potency [25], and it has also been suggested that there are specific advantages to covalent attachment of these peptides over that of physical adsorption, including controllable concentrations and directed peptide conformations, which will affect cellular interaction. A non-eluting device would also allow for a reduced medical device classification, and is therefore of significant clinical interest [26]. The use of peptides to improve bone tissue regeneration was reviewed by Wang et al. [27], who also covered attachment methodologies and the general progress in the area. The authors highlighted the need for much more work in the field, with the need for systematic studies to more strongly elucidate the effects of specific peptides and the preparation methods used for these modified alloplastic grafts.

Therefore, in this review, we propose an overview of the different strategies proposed in the literature to design peptide-enhanced bone graft substitutes and a critical perspective of the peptide selection and role of attachment methodology on their clinical efficacy. Hence, the scientific community and researchers in the tissue engineering field can get insights into the design and manufacture of bioactive graft materials able to efficiently regenerate bone.

## 2. Peptides

Peptides, short amino acid chains that are generally copied from active sites in larger protein molecules, offer an approach to overcome problems associated with orthobiologics while retaining the osteogenic effects. They mimic the signalling and/or the binding domains of the larger proteins, which offers a potential solution to problems with steric effects, immunogenicity and susceptibility to degradation.

Steric effects and folding problems represent a major issue. In response to interaction with a material, bone proteins will fold in certain manners, determined by the specific properties of the material [28]. This folding is highly specific, and therefore difficult to control, manipulate and can often be random [25]. It can lead to the signalling and binding domains of the large protein becoming sterically unavailable for cellular interaction [25]. To overcome this unavailability, high dosages are used, which are inherently difficult to control and, as mentioned previously, often result in adverse effects [22]. In comparison, bound peptides have a higher stability and undergo highly controllable determined folding, making them available for cellular interaction, and therefore dosage control is significantly less difficult. Peptides can alleviate the issues surrounding steric effects associated with larger proteins, and even have an increased repeatable cellular signal [29].

Immunogenicity is also a problem, as producing proteins via recombinant human expression carries a high economic cost, as they inherently carry the risk of immunogenicity, which must be removed through expensive purification and quality controls [30]. The FDA approved BMP modified bone grafts, as an average, cost approximately four times that of their non-modified alloplastic bone graft alternatives [4]. Peptides can be fabricated synthetically using liquid or solid-state phase synthesis, which offers no risk of immunogenicity and a significantly reduced cost through reduced material wastage, and processing and purification times [25,30].

There are also potential issues in the long-term stability of proteins. Subtle changes in pH and/or temperature during production, storage, implantation or within the human body can cause the irreversible denature of proteins [31,32]. This sensitivity is related to their functionality and structure (secondary, tertiary and quaternary folding) being destabilised through the breakage of weak hydrogen bonding. Peptides are significantly more resistant to pH and/or temperature changes as their function is not impaired unless covalent bonds are broken [30]. Once again this allows peptides to alleviate another problem associated with large proteins, and their resistance opens up potential immobilisation routes not available to proteins [29].

There are a vast array of proteins associated with bone and its regeneration. Due to the quantity of different proteins and large number of possible cellular interactions there are many specific peptides of interest. This number is increased further by the possibility of combinations and variations based around a single peptide sequence. It is therefore understandable that most interactions between peptides and cells are yet to be fully understood. In 2016, there were detailed reviews published around utilising various bioactive peptides for bone tissue regeneration, which provides evidence that there is currently significant growing interest in this area. Of these reviews, the state of the art was covered concisely by two groups: Pountos et al. [33] and Visser et al. [34]. With the number of peptides, reported to enhance bone tissue regeneration, growing and compounded by the various modifications of each peptide available, there is an increasing need to compare peptides in terms of bioactive capability. The reviews published by Pountos et al. and Visser et al. did not facilitate this important step.

To better understand current knowledge, a critical review of the numerous published peptide investigations related to bone tissue regeneration was performed. Due to the large number of variables concerning research of this nature (in vitro cell types used, number of cells, in vivo studies, possible peptide immobilisation routes, cell behaviours measured and base graft material to name a few), it is not possible to compare peptides quantitatively. However, the studies can be categorized through both attachment methodology and peptide grouping to allow for comparison.

## 3. Attachment Methodology

The attachment of peptide to substrate is important. Most of the proteins and peptides currently being utilised for stimulating osteoinduction in allografts, xenografts or alloplastic bone graft substitutes are soluble. This solubility leads to an immediate dissipation upon implantation at the targeted delivery site, resulting in a reduced efficiency and potency of the bioactive molecule on the targeted tissues [4]. These specific biomolecule inclusions can be troublesome elsewhere in the body, often disturbing homeostasis. There is evidence showing certain peptides can inhibit cell adhesion, hinder bone formation, stimulate unwanted bone resorption and impair cell metabolic activity [32,35,36]. As such, peptides are often conjugated to substrates in order to enhance, sustain and control the desired effects.

A variety of methodologies are used for this purpose. These range from simple adsorption to more complex, multi-stage chemical processes. To prevent the rapid biodegradation of the substrate, a large proportion of biomaterials used in bone tissue regeneration are relatively chemically inert with unreactive species presented at the surface. Therefore, to attach specific biomolecules, a functional group is often required for the more complex methodologies. Aminosilane chemistry and plasma polymerisation are commonly used for this process [26,37,38,39,40,41,42,43,44,45]. These methodologies can also introduce more control over dosage and release rate, which is important, as it has been shown, for example, that high concentrations of RGD, a commonly used peptide, can be detrimental to cell adhesion [33,46]. Careful consideration when choosing attachment methodology is therefore important.

**Figure 1:**
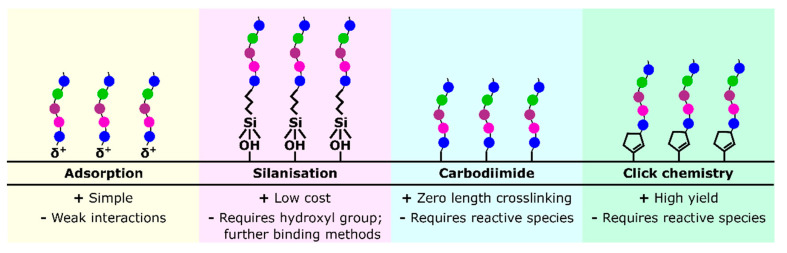
A diagram representing four common peptide attachment methods: adsorption, silanisation, carbodiimide crosslinking and click chemistry.

As there is a wide diversity of methodologies presented in the literature, with specificity depending on peptide and substrate selection, this review focussed on four broad attachment methodologies regularly used to allow for comparison, and to summarise the more general advantages and disadvantages of these methods: adsorption, silanisation, carbodiimide crosslinking and click chemistry, represented in Figure 1, as well as hydrogel incorporation, another commonly used peptide delivery method.

### 3.1. Adsorption

Most of the existing research in this area has involved passive surface adsorption of peptides as the chosen delivery method. For this methodology, substrates are simply incubated in solution of the chosen peptide [37,38,47]. In physical adsorption, the proteins or peptides are held in place by weak ionic interactions and/or van der Waals forces [26].

Adsorption sometimes relies on the alteration of the originally designed peptides by the addition of amino acid sequences, with a specific affinity for the chosen carrier material (e.g., polyglutamate, polyaspartate sequences, non-canonical amino acids such as γ-carboxyglutamate or phosphoserine, all of which show a specific affinity for calcium phosphates) [37,38,39,48]. This could have an effect on the peptide, for example, the additional amino acids can impact the secondary structure of the peptide, thus interfering with cellular interactions [38]. However, the extent to which these affinity sequences affect the efficiency of the signalling peptide are still disputed in the literature [38,45].

Adsorption has been used with many different peptides and substrates, allowing for improved osteogenic properties both in vitro and in vivo. However, adsorption requires certain considerations. While technically simple, this system is plagued with complications such as: ectopic dispersion, surface dispersal, concentration control and peptide conformation/orientation [26]. The release of the peptide is often not examined in the literature, however when measured it is high. Saito et al. [43] noted 50% release over 14 days, and Lin et al. [49] and Li et al. [50] saw 100 and 60% release over 4 days, respectively. With the concerns over ectopic bone formation and previously mentioned high dose issues, this high level of release could potentially cause problems in vivo.

### 3.2. Silanisation

One method which allows for peptide conjugation is aminosilane chemistry or silanisation. This is a technique used to generate functional groups at the surface of materials for the attachment of peptides [45]. Surfaces which contain hydroxyl groups, such as glass, are functionalised with alkoxysilanes, leaving a surface which can then be further functionalised through polymerisation. While not specifically used to attach peptides to substrates, a large section of the literature has used this method to functionalise surfaces to allow for peptide conjugation.

During silanisation, the surface is first cleaned and oxidised using an oxidising agent, such as piranha solution, to leave the hydroxyl groups required [51]. The activated surfaces are then placed into an alkoxysilane, either solution or atmosphere, where silanisation occurs, attaching the siloxane to the surface. The head of the siloxane is reactive, which allows for covalent attachment of the desired functional group.

The technique has been shown to be nontoxic and repeatable in many in vitro and in vivo investigations, and has therefore been used to allow for the binding of osteogenic peptides with high success [26,44,45,52,53].

However, there are disadvantages associated with this functionalisation technique when compared to others. Firstly, silanisation is a method of surface modification, and a further step is required to bind the peptides to the surface, which can vary in difficulty [54,55,56]. As silanes require an oxidised surface, this can be a limiting factor in material selection [57]. Silanisation processing conditions vary, with some requiring 120 °C heat for 4 h [45], which could be problematic on a substrate containing organic components. There are also reports which indicate the silane layer, generated in the grafting of amino groups, can significantly diminish the original osteoconductive properties of the biomaterial, and also negatively affect the pH of in vitro cultures [58,59].

### 3.3. Carbodiimide Crosslinking

If a surface has a suitable reactive species, either through substrate choice or surface modification, chemical conjugation is possible. One method of chemical conjugation is using carbodiimide crosslinker chemistry, which allows for crosslinking with an amide bond. Other similar functional groups can be used for a similar bond, but carbodiimides have advantages as they allow for zero-length crosslinking, preventing the need for additional atoms which can negatively affect cellular interaction, have mild aqueous reaction conditions, which include ambient temperatures and pressures, and produce relatively harmless by-products which are easily removed through washing steps [60]. Various in vitro and in vivo studies have shown that carbodiimide generated crosslinks are not cytotoxic and are resistant to enzymatic digestion, and demonstrated success covalently binding collagen to substrates [61,62,63]. This makes them suitable candidates for the conjugation of osteogenic peptides.

There are three main carbodiimides used, dicyclohexylcarbodiimide (DCC) and *N*,*N*’-diisopropylcarbodiimide (DIC) and 3-(dimethylaminopropyl)-3-ethylcarbodiimide (EDC). Both DCC and DIC are allergens and insoluble in water; EDC carries neither of these disadvantages is therefore the most commonly used [64]. EDC reacts with carboxyl groups to form an active O-acylisourea intermediate, which is then displaced by nucleophilic attack from the primary amino groups of peptides, forming an amide bond and isourea as a by-product. However, carbodiimide crosslinking can have issues. O-acylisourea can undergo hydrolysis, recovering the system to the unreacted state, which is particularly problematic due to the high amount of water present in the aqueous reaction conditions [65]. With a large excess of EDC in the reaction system, there can sometimes be the formation of N-acylisourea as a side reaction [66].

Succinimidyl esters are often employed to help alleviate these issues. The use of N-hydroxysulfosuccinimide (NHS) produces a less hydrolysis sensitive intermediary compound allowing for more efficient peptide conjugation [65]. The addition of NHS also prevents the formation of N-acylisourea since the succinimidyl ester cannot undergo displacement. The addition of NHS causes the potential formation of a different by-product, sulfo-β-alanine, which is also reported to be easily removed by washing steps and has a relatively low toxicity [66].

Carbodiimides also have potential drawbacks, however. One negative complication of EDC peptide conjugation is that certain amino acids can potentially react with the EDC in side-reactions (e.g., histidine, tyrosine and cysteine) which reduces conjugation efficiency, however these issues can be combatted through different specific means, dependent on the peptide chosen [66]. This makes them a promising choice for peptide conjugation.

### 3.4. Click Chemistry

Click chemistry is a term used to describe a range of chemical reactions for molecule conjugation. Click reactions are defined by certain properties, all of which make them advantageous in surface conjugation [67,68,69]. They have a high yield, producing low levels of ideally inoffensive by-products. They take place in ambient conditions, and are exothermic, with a thermodynamic driving force toward a single reaction product. These benefits have therefore seen them used for the conjugation of peptides to substrates, with some use in the osteogenic field [70,71,72,73,74,75].

In the most common click reaction, the copper-catalyzed azide-alkyne substitution (CuAAC), compounds with azide and alkyne end groups are reacted in the presence of a Cu(I) catalyst, with a 1,3-dipolar cycloaddition forming a 1,2,3-triazole bond [68,69,74]. This process has advantages over other conjugation methods as the bonds have stability in aqueous conditions and suitability for long term storage.

However, click chemistry has problems. The initial reactive species required for the click reactions mean that both the peptides and the surface may need modification to give them the specific endgroups to allow for the reactions to take place [70,71]. The reactions themselves also each have potentially negative considerations. For example, with the CuAAC, the copper introduced into the system which is difficult to remove and can cause toxicity in vivo [72]. The use of some azides may also be problematic at larger scales due to their inherent reactivity and explosivity [73]. Other click reactions are based around thiols, which are sensitive to oxidisation that leaves them unreactive, and this therefore must be considered when designing the reaction [74].

### 3.5. Hydrogel Incorporation

While some articles have peptides conjugated to the surface of hydrogels, others have instead incorporated the peptide within their hydrogel and seen generally positive results, both in vitro and in vivo [29,76,77,78,79]. Hydrogels have high cytocompatibility and a low inflammatory profile, and their mechanical properties can be tailored during production [78]. Despite these benefits, there are properties of hydrogels which may make them unsuitable for osteogenic use. The mechanical strength of hydrogels is generally lower than that of both bone and other bone graft materials, which may limit their clinical use, and the degradation rate of hydrogels must be well considered during design, in order to ensure a sustained release of the incorporated peptide and thereby avoid potential ectopic effects [80].

## 4. Peptide Selection

Alongside attachment methodology, the selection of peptides provides a method to control the efficacy of an alloplastic bone graft, with choice and specific sequence both playing a role in the overall success. Many different peptide groupings and sequences have been tested in literature. A few select peptide groupings were selected and discussed further based upon their significant impact in the research and clinical sectors: RGD, PHSRN, FHRRIKA, KRSR, GFOGER, P15 and the BMP mimetic peptides. In order to compare these peptides, the studies are summarized through the peptide grouping, specific amino acid sequence, attachment methodology, substrate material, and if there was or not a significant increase or decrease to cell attachment, spreading, differentiation and matrix mineralisation of a bone specific cell type in vitro, and any significant differences shown in vivo.

### 4.1. RGD Peptide

With approximately 90 wt.% of organic bone matter consisting of collagen type I, a large selection of peptides have been isolated from this protein and investigated for specific bioactivity (examples include RGD, GFOGER, DGEA, BCSPTM-1 and P15) [33,34]. The most comprehensively researched peptide is RGD (Arg-Gly-Asp), first discovered by Pierschbacher et al. in 1984 [81]. This sequence is part of a ligand found in many bone matrix proteins such as fibronectin (FN), vitronectin (VN) and collagen [82]. Attachment to RGD and many other similar adhesion peptides is mediated through cell surface receptors, found in the integrin superfamily, recognition and interaction. However, there is a redundancy in integrin affinity for adhesion peptides which leads to many cells possessing the same integrins, and as a result may cause non-specific cell attachment to certain common adhesion peptides [83].

There are many publications regarding the development of bone-related materials coated with RGD based peptides, shown in Table 1, commonly using adsorption to attach them to substrates, with studies consistently reporting improved osteogenic cell attachment and spreading in vitro [29,54,76].

RGD does not always increase cellular adhesion and spreading, however. A longer chain RGD (GPenGRGDSPCA) was adsorbed to hydroxyapatite and examined in studies from Hennessy et al. [37] and Sawyer et al. [38]. While human MSCs (HMSCs) were able to attach to the material, cell spreading was poor. It was theorised that while RGD is useful as it provides a cellular binding site, on a material such as hydroxyapatite, which already absorbs proteins that themselves provide a stronger binding site (e.g., FN, VN), the RGD may competitively bind cells and therefore lead to an overall weaker attachment [37]. Sawyer et al. [38] hypothesised that the lack of effect may be due to the adsorption of the peptide itself, and recommended a covalent attachment method—however noted that this may alter useful surface properties. It is therefore important to consider the substrate alongside the desired peptide in order to optimise the osteogenic properties for use in bone graft applications.

RGD has been used in vivo for bone regeneration. Zhang et al. [52] attached RGD to hydroxyapatite through silanisation and demonstrated improved bone healing when implanted into the femoral condyles of rabbits, although little discussion as to why RGD had this effect is included. A recent in vivo study performed by Scholz et al. in 2013 [94] comparing spinal fusion in sheep treated with either cyclic RGD or rhBMP-2, indicating a similar effectiveness in promoting bone regeneration, with the three-dimensional structure of cyclic RGD thought beneficial through increased receptor affinity. Pan et al. [92], however, found a longer chain version of RGD had limited results in vivo, with rabbit femoral defects only healing when transforming growth factor β (TGF-β) was included into their scaffold. These limited effects have also been highlighted in several studies which examined RGD peptide efficacy further than attachment and spreading.

While further cellular effects of RGD have been studied, with increased osteogenic differentiation and matrix mineralisation reported [47,55,70,89], there is a lack of consensus on these within the literature, and Pan et al.’s observation that additional inclusions are required to allow for the RGD improvement is a common occurrence. Bilem et al. [54] and Dee et al. [87] both attached an RGD peptide to glass coverslips through silanisation. Dee et al. saw no effect on osteoblast adhesion and hypothesised that, while RGD allows for integrin binding, this alone may not be good enough to increase attachment and a more complex methodology would be more suitable. Bilem et al. [54] examined human MSC attachment to the surface, and witnessed attachment and spreading. They did not, however, encounter osteogenic differentiation, though the cells’ stemness was removed. They hypothesised that while RGD was useful for cellular attachment, osteogenic markers or factors were required to fully induce osteoblastic differentiation. This ties in to work from Moore et al. [71] who examined RGD attached to SAMs through click chemistry, and found HMSCs did not differentiate. Moore et al. theorised that this was due to a lack of osteogenic medium, highlighting that where MSC differentiation had been triggered by RGD in He et al.’s study [70], osteogenic media was used. The use of osteogenic medium must therefore be taken into account when assessing the results of in vitro studies with MSCs, with the other literature also pointing to a positive RGD differentiation effect also including specific osteogenic supplements in their media [93]. A study by Shekaran et al. [78] demonstrated increased differentiation and proliferation from an RGD peptide, however in their case this was part of a hydrogel which contained BMP-2, another osteogenic inclusion. These studies demonstrate that whilst RGD is usually capable of increasing cellular adhesion, RGD alone does not cause increased osteogenesis, and there are more factors to consider when designing a peptide-enhanced bone graft material.

### 4.2. PHSRN Peptide

PHSRN (Pro-His-Ser-Arg-Asn) is a peptide derived from fibronectin, and though it is not effective alone, it has been demonstrated to have a synergistic effect with RGD to enhance cellular activity through allowing for α_5_β_1_ integrin binding [47,93]. There is a range of the literature covering the use of these peptides together, shown in Table 2, however with mixed results.

**Figure 2:**
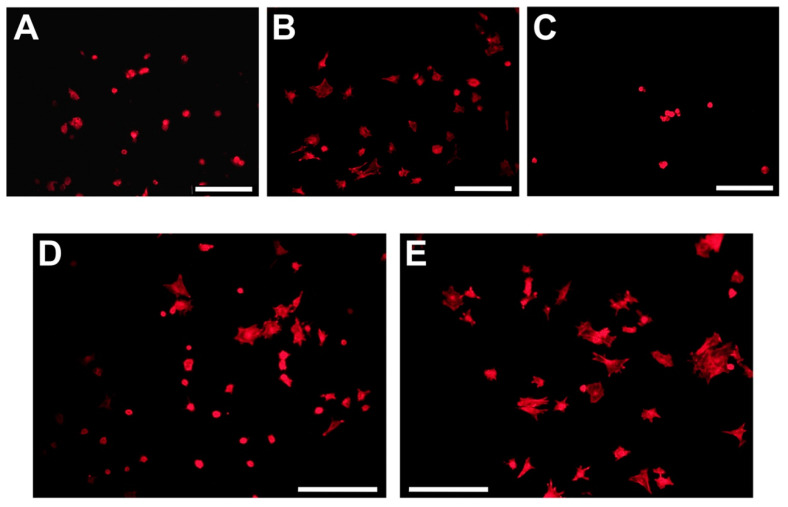
Spreading of Saos-2 cells on biofunctionalized surfaces after 4 h of incubation. (**A**) Ctrol, (**B**) RGD, (**C**) PHSRN, (**D**) MIX, and (**E**) Platform. Images were acquired by fluorescence microscopy and show only staining of actin filaments with phalloidin-rhodamine. Scale bars: 200 μm. Reproduced with permission from [47].

In a study by Paredes et al. [56], RGD and PHSRN did not improve rat bone MSC spreading on titanium surfaces, with RGD alone performing better in their tests. They concluded this was due to RGD and PHSRN working similarly through providing sites for α_5_β_1_-mediated adhesion and instructing cells to adhere, therefore making a combination redundant. This contradicts work from others who have combined RGD and PHSRN in more complex ways and demonstrated synergistic effects. Mas-Moruno et al. [47] compared RGD and PHSRN alone to a mixture of the two peptides immobilised to the surface of titanium. Alongside this, they also created a dimeric platform containing both peptides together, with a spacing between the peptides mimicking that found in natural fibronectin. The mixture of peptides did not increase SAOS-2 (an osteoblast-like cell line) cellular spreading and proliferation compared to RGD alone, however the dimeric platform did. This data is shown in Figure 2, and indicates that using peptides in a conformation more similar to nature is highly beneficial. Neither the mixture nor platform significantly increased osteogenic differentiation, however, whereas RGD alone did, although this was linked to the cells exposed to combination peptides having higher proliferation and therefore reduced phenotype expression.

A combination of RGD and PHSRN which more closely mimics their natural confirmation was used by Benoit et al. [29], with a longer chain RGD allowing for larger spacings between the peptides. Attachment, spreading and cell differentiation were improved compared to RGD alone. Matrix mineralisation decreased, thought to be due to the peptide being recognised by the cells as extracellular matrix (ECM), and therefore down-regulating ECM production. The improved attachment, spreading and differentiation were thought to be not only due to the synergistic effect of RGD and PHSRN, but also the extended RGD chain mimicking the spacing between RGD and PHSRN found in nature, again highlighting the benefits to efficacy that can be delivered through a more considered biomimetic approach. The synergistic effect of the inclusion of PHSRN with RGD improved results, particularly when the peptides were used in a more natural configuration. However, overall, the the literature highlights a lack of potential for osteogenic effects from these peptides, and as such, other peptides have been studied.

### 4.3. FHRRIKA and KRSR Peptides

Osteoblast cell attachment is not only mediated through integrin binding, but also by the interaction of transmembrane heparan sulphate proteoglycans with heparin binding sequences found within many different proteins associated with bone tissue. These types of additional interactions are also believed to significantly impact osteoblast attachment behaviour, as heparan sulphate has been detected immunohistochemically on the membranes of osteogenic cells attached to formed bone matrix [87,97]. Based on their promising publication history and complementary action, FHRRIKA and KRSR have potential for future clinical impact, and studies using these peptides are shown in Table 3.

**Figure 3:**
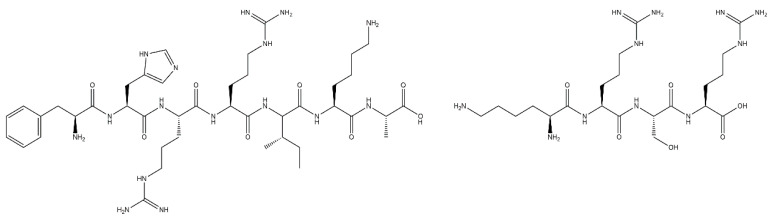
The chemical structure of FHRRIKA (left) and KRSR (right) peptides.

First reported in 1999 by Rezania and Healy [55], FHRRIKA (Phe-His-Arg-Arg-Ile-Lys-Ala) is a peptide isolated from the heparin binding domain of BSP (Figure 3). This peptide is reported to significantly enhance cell proliferation, spreading, and in some cases, matrix mineralisation [38,98,99,100]. Rezania and Healy functionalised quartz discs with FHRRIKA through silanisation and showed that FHRRIKA modified surfaces significantly enhanced cell spreading, however saw little effect on mineralisation [55]. FHRRIKA enhanced cell attachment and spreading were in a paper published by Paredes et al. [56], on titanium surfaces functionalised via silanisation. Further study is required to build upon these promising results.

KRSR (Lys-Arg-Ser-Arg), is also a heparin binding peptide, present in bone sialoprotein, FN, VN, osteopontin and thrombospondin (Figure 3) [100]. It was first reported by Dee et al. [87] in 1998, where it was shown to selectively enhance osteogenic cell adhesion on borosilicate glass functionalised using aminosilane chemistry. Dee et al. also hypothesised KRSR may be subject to binding through α_v_β_5_ integrin receptors, alongside the heparin binding sequence, further increasing attachment. Osteoblast cells are known to express this integrin, and therefore this peptide has high potential in the osteogenic field, which has been explored in the literature.

The selective osteoblast attachment has also been demonstrated by Hasenbein et al. [85] though the micro patterning of KRSR on borosilicate glass using elastomer stamps. This selective attachment gives KRSR an advantage over non-specific peptides, as it allows for the direct targeting of bone cells. Like RGD, KRSR has demonstrated consistent success in allowing for attachment and spreading, with various attachment methodologies and substrates [38,41,79,84,88,101]. However, Dettin et al. [84] found that RGD increased osteoblast attachment to a higher degree than KRSR, using different attachment methodologies and substrates, though did not explain this difference. Anderson et al. [102] witnessed a similar effect in a study using HMSC cells, and this potential limitation of KRSR requires further investigation.

In a report published by Gentile et al. [98] FHRRIKA and KRSR peptides were attached to polyhedral oligomeric silsesquioxane nano particles, functionalised through plasma polymerisation of acrylic acid and subsequent carboxyl activation via EDC/NHS. In this work the peptides did increase the attachment of MSC cells, and FHRRIKA peptides resulted in a significant increase in alkaline phosphatase (ALP) production, indicating the differentiation of the cells, though KRSR did not increase differentiation more than plasma treatment alone. This lack of consistency present in the literature is an area where more study is required.

Whilst both FHRRIKA and KRSR have demonstrated success in enhancing the osteogenic properties of materials, study has been relatively limited and further work is required to fully elucidate their cellular effects across the different cells that contribute to bone regeneration, and their osteogenic capacity in vivo before widespread use [103].

### 4.4. GFOGER Peptide

GFOGER (Gly-Phe-Hyp-Gly-Glu-Arg) is another peptide derived from collagen, specifically from the α_1_ chain of collagen-1. When manufactured, Gly-Pro-Pro ends are often included, which allow the peptide to take up a collagen-mimicking triple helical structure, which is essential for α_2_β_1_ integrin binding, part of the peptide’s mechanism of action. This binding enhances development and expression of an osteoblastic phenotype and is therefore important for differentiation, and it is also has been demonstrated to improve matrix mineralisation [104]. GFOGER has an advantage in osteogenic properties over native collagen, as other competitive binding sites are found on native collagen, but not present on the GFOGER sequence, which allows for a more specific effect [105]. This specific osteogenic effect allows for advantages in osteogenic applications over entirely adhesive peptides, such as RGD [78], and several studies have investigated its use in these applications.

GFOGER has been used successfully to increase cellular attachment, osteogenic differentiation and matrix mineralization of stem cells, and has also demonstrated success in vivo in bone healing studies [78,93,104,105,106]. These studies have involved a variety of attachment methodologies and substrates, shown in Table 4.

**Figure 4:**
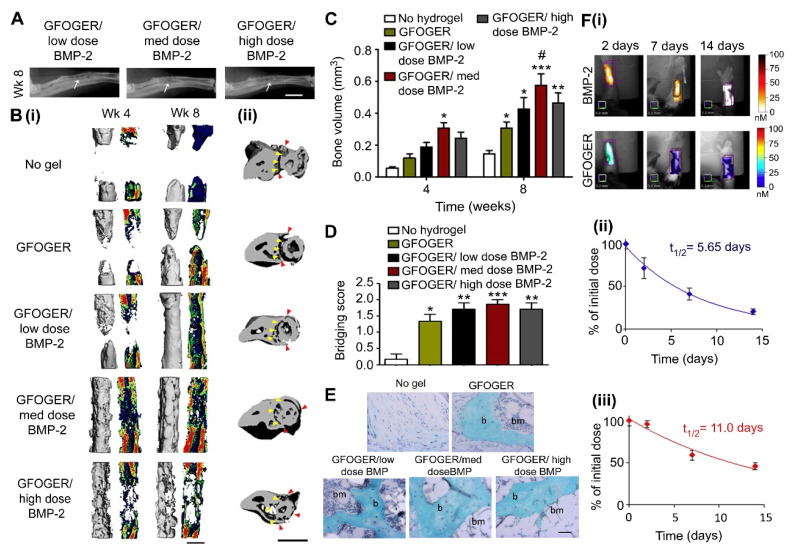
GFOGER-functionalized PEG hydrogels with low dose BMP-2 bridge radial segmental defects without altering ulnar structure. (**A**) Radiographic images, white arrows indicate space between ulna and radius which is not present in the high BMP-2 dose image, scale bar 2 mm. (**B**) 3D μCT reconstructions of (**i**) radius in sagittal view (**left**) with mineral density mapping (**right**), and (**ii**) radius and ulna in transverse view. Yellow arrowheads indicate boundary between the ulna and radius prior to implantation, red arrowheads indicate the position of the ulna closest to the radius at 8 weeks, scale bar 1 mm. (**C**) μCT measures of bone formation, *n* = 6–7. (**D**) Scoring of defect bridging at 8 weeks, *n* = 6–7. (**E**) Sections stained with Safranin-O/Fast Green at the center of defect, scale bar 50 μm; b—bone, bm—bone marrow. (**F**) (**i**) Representative FMT images and FMT quantification of % implanted dose retained in radial defect space over time in vivo for (**ii**) high dose BMP-2 labeled with Vivotag 800 and (**iii**) GFOGER peptide labeled with Vivotag 680, *n* = 6. * *p* < 0.05, ** *p* < 0.01, *** *p* < 0.001 compared to defect receiving no hydrogel implant, # *p* < 0.05 compared to GFOGER hydrogel. Reproduced with permission from [78].

One such study was performed by Shekaran et al. [78], who demonstrated that polyethylene glycol-BMP-2 hydrogels modified with surface GFOGER could increase cell spreading and osteogenic differentiation in BMSCs, in comparison to an RGD modification. When studied in vivo, they demonstrated that GFOGER could promote healing even without the inclusion of the BMP-2 component of the hydrogel, shown in Figure 4. These results highlight the potential of GFOGER for use in osteogenic applications.

Hennessy et al. [37] adsorbed GFOGER to the surface of hydroxyapatite disks, however found GFOGER was unable to increase cell attachment and decreased cell spreading, and were unable to explain this issue. Wojtowicz et al. [105] commented on this and claimed this may be due to a difference in peptide sequencing, as they showed positive results in a similar study. This demonstrates the importance of peptide selection, even within one peptide grouping, with a peptide which can more closely mimic the natural protein favourable. The nature-mimicking conformation of GFOGER is therefore a promising peptide for use in this field.

### 4.5. P15 Peptide

**Figure 5:**
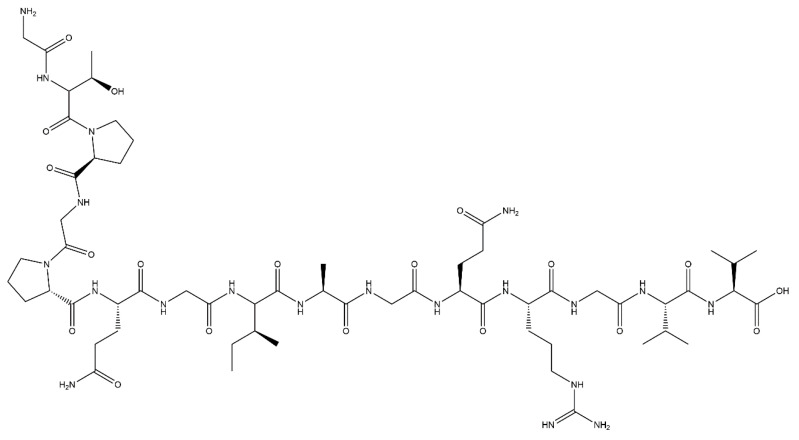
The chemical structure of P15 peptide.

P15 is again a peptide based upon a sequence of collagen (Gly-Thr-Pro-Gly-Pro-Gln-Gly-Ile-Ala-Gly-Gln-Arg-Gly-Val-Val) (Figure 5). It is a high affinity ligand found in the α_1_ helix of collagen type I, 766–780 residues, which interacts with a specific collagen binding cell surface integrin (α_2_β_1_) [37]. First reported by Bhatnagar et al. in 1997 [107], the P15 peptide has been shown in vitro and in vivo to enhance osteogenic cell attachment and proliferation, and in some more recent publications has also been shown to stimulate MSC differentiation [108,109]. When P15 is present at the surface of a material it functions as a substitute for collagen type I. The binding of cell surface integrins (α_2_β_1_) to P15 leads to the production of growth factors and cytokines, which in turn stimulate matrix production and mineralisation [110]. As the integrin α_2_β_1_ is known to be collagen specific, it is believed that this interaction would not compete with other cell surface integrins for FN (α_IIb_β_3_ or α_5_β_1_) or VN (α_v_β_3_), proteins found in blood [37]. P15 interaction is RGD independent, whereas VN and FN both bind integrins through RGD mechanisms [37].

Of the collagen-based peptides mentioned in Section 4.1, P15 is the only sequence currently FDA approved for use in either periodontal defects, PepGen P-15^™^ (Dentsply©) or cervical disc reconstruction, i-Factor^™^ (Cerapedics©). P15 has also received significant attention as it interacts with cells through a specific integrin, and therefore does not compete with other protein interactions [37]. This sequence is supported by a large body of in vitro studies and clinical trials, and carries specific commercial value [11,12,18,110,111,112,113,114,115,116]. As P15 is currently available on the market, for dental (PepGen^®^ (Dentsply©) and orthopaedic (i-Factor^®^ (Cerapedics©)) applications, there is a large body of published literature surrounding its successful use, with some shown in Table 5.

Studies have investigated P15 adsorbed to bovine bone [107,108,114]. Bhatnagar et al. [108] showed that P15 stimulated matrix mineralisation and cell differentiation, but also alluded to problems surrounding peptide dissolution and concentration. Interestingly Bhatnagar et al. also showed that like KRSR, P15 can inhibit fibroblast adhesion, which increases its relevance to the osteogenic field [107]. Nguyen et al. [114] showed the beneficial effects of P15 persisted even when coated bovine bone was immobilised in an inert hydrogel carrier, and similar effects have been demonstrated with titanium surfaces modified through silanisation by Liu et al. [53]. These results demonstrate the high potential of P15 in the field, with the bone cell specificity of KRSR but with a strong backing of data evidencing its efficacy in in vitro and in vivo studies.

There have been several studies demonstrating the efficacy of P15 in vivo. In animals, ANKYLOS^®^ (Dentsply©) and i-Factor^®^ (Cerapedics©) significantly enhanced bone regeneration in alveolar bone in dogs and the tibia of osteoporotic dogs, respectively, in studies from Pedersen et al. [110] and Schmitt et al. [118]. However, Pedersen et al. also indicated that, in normal rats, bone regeneration was not accelerated when treated using i-Factor^®^. This is a result that is important to note, whilst bearing in mind the major clinical challenge of orthobiologics is to provide healing in compromised patients.

Human studies have also been performed in different indications. As part of a large multi-centre trial, Yukna et al. [111] published the six month findings of human patients with periodontal osseous defects treated with a P15—bovine bone graft material. The study indicated that bone regeneration was enhanced in the presence of P15, which potentially also had a knock-on effect in stimulating enhanced regeneration of the periodontal ligament. Anterior lumbar interbody fusion using P15—bovine bone (i-Factor^®^ (Cerapedics©)) was investigated in 110 human patients in a published study by Mobbs et al. in 2014 [112]. In this study, it was concluded that the P15 substitute demonstrated similar clinical outcomes to patients treated with either autograft or Infuse^®^ (Medtronic©). However, a clinical evaluation published by Kasaj et al. [119] indicated no significant differences between groups treated by traditional means and those treated with P15—bovine bone in a hydrogel carrier. These results may be related to peptide dispersion, as here P15 was adsorbed to the surface of the xenograft and not covalently anchored.

There are numerous other clinical studies involving bone regeneration stimulated through the use of P15, which can be found detailed in a review published by Pountos et al. [33]. To summarise, bone tissue regeneration augmented with P15 was shown to be effective in a number of different indications, and has demonstrated the ability to improve regeneration in a compromised healing model. These results, and the market approval for existing P15 based products, make it an attractive solution.

### 4.6. BMP Mimetic Peptides

Peptides derived from BMPs have also seen use in this field, with the literature generally focused on the BMP-2 mimetic peptide, also known as P24 (Ser-Lys-Ile-Pro-Lys-Ala-Ser-Ser-Val-Pro-Thr-Gly-Leu-Ser-Ala-Ile-Ser-Thr-Leu-Tyr-Leu-Asp-Asp-Asp), however with some use of BMP-7 and -9 mimetic peptides. These peptides contain the knuckle epitope of the protein, and bind to BMP receptors as their mechanism of action, and are 20+ amino acids in length. BMP mimetic peptides have shown universally positive results across a variety of attachment methodologies and substrates, shown in Table 6, with several also demonstrating in vivo efficacy by inducing ectopic bone formation in rodents [49,120,121].

Although they are effective, BMP-2 and its associated peptide have a short retention time in the body, reducing their efficiency [49]. For this reason, research has focused on defining suitable substrates and attachment methodologies to prolong the release of the BMP-2 mimetic peptide to allow for sustained osteogenesis.

**Figure 6:**
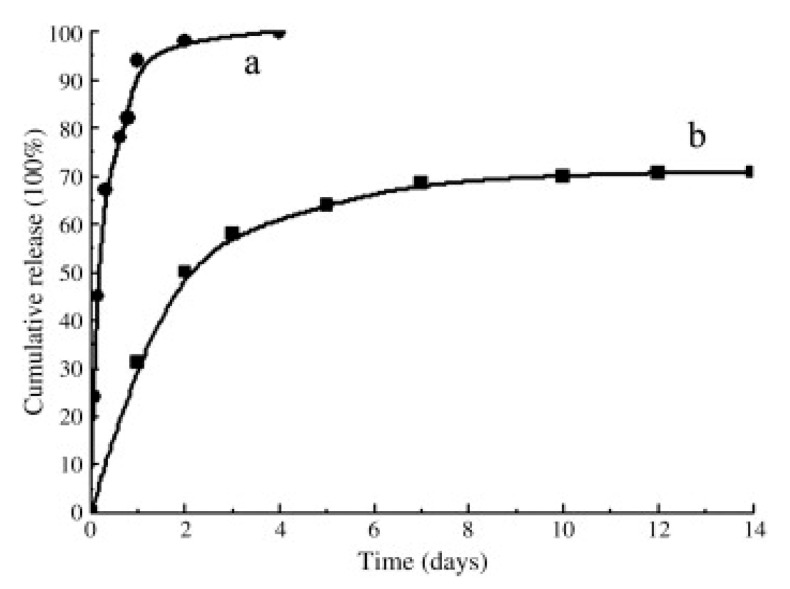
The profiles of P24 peptide released from PLGA-(PEG-ASP)n scaffold in the absence (**a**) and presence (**b**) of NHS/EDC. Reproduced with permission from [49].

Research has investigated both simple and complex methodologies to achieve the necessary attachment. Niu et al. [42] encapsulated their peptide into chitosan microspheres, before blending these into a polymer, which slowed the full release of the encapsulated peptide to approximately 18 weeks. Lin et al. [49] compared adsorption to EDC-NHS mediated covalent binding of BMP-2 mimetic peptide to a PLGA-(PEG-ASP)n polymer. In their study, the use of EDC-NHS slowed the release rate drastically, altering the release from 100% within 4 days to only approximately 70% over 14 days, shown in Figure 6, and showing ectopic bone formation over 12 weeks in vivo. While Li et al. [50] only adsorbed BMP-2 mimetic peptide to a bone ceramic, with the surface mineralised through use of simulated body fluid, they also exhibited a release profile similar to that of Lin et al.’s covalently bound peptide, with a partial release over 14 days and efficiency in vivo over 12 weeks. They posited that due to the BMP-2 mimetic peptide ending with a repeating Asp sequence, this gave the peptide a high binding affinity for apatite-based materials, and therefore would allow the peptide to bond to the scaffold without the need for more complex methodologies. These studies highlight that consideration of substrate and binding methodology when designing a peptide-enhanced bone graft material can allow for greatly improved bioactive properties.

As well as studying RGD, both Moore et al. [71] and He et al. [70] attached BMP-2 mimetic peptide to a substrate using click chemistry and investigated the effect on BMSCs. As discussed in Section 4.1, Moore et al.’s SAMs saw attachment and differentiation, but no improved mineralisation, and suggested more specific biological signalling may be required to upregulate this process. He et al. cultured their hydrogel in osteogenic medium and saw attachment, differentiation and mineralisation, demonstrating the benefits of this specific signalling. Moore et al. also studied a combination of RGD and BMP-2, and found that, even without osteogenic medium, the combination of the peptide allowed for increased mineralisation, with it hypothesised that RGD increased cellular adhesion and therefore enhanced the amount of interaction of cells with the bound BMP-2. This combination effect is a commonly reported phenomenon and has warranted further study.

### 4.7. Combinations of Peptides

As the peptides discussed have different mechanisms of action, they are often used in combination to increase their effects, shown in Table 7. As studies of RGD have found diminished effects, the use of RGD in conjunction with other peptides, including FHRRIKA and BMP-2 mimetic peptides, has been investigated. These methodologies allow for a more specific, tailored approach where peptides with separate mechanisms can increase the osteogenic properties, compared to either peptide alone. There is a growing body of evidence that this more tailored approach is beneficial.

The synergistic effect of RGD and BMP noted by Moore et al. [71] in Section 4.6 has also been noted in the other literature, with RGD thought to increase adhesion while the BMP mimetic peptide increases the osteogenesis of MSCs [54]. This effect has been seen with BMP-2 mimetic peptides by Bilem et al. [54], and Zouani et al. [90] who also saw the same synergy with RGD and BMP-7 and BMP-9 mimetic peptides.

RGD and KRSR both promote cellular attachment, however through integrins and proteoglycans, respectively. These binding mechanisms can be used in conjunction to increase attachment further. Dee et al. [87] examined the attachment of osteoblasts onto glass surfaces to which peptides had been attached through silanisation. When coated with RGD alone, cells attached but no more than the control glass. KRSR alone increased the attachment. When both RGD and KRSR were used attachment was increased further, due to this dual binding mechanism. Rezania and Healy [55] demonstrated that a longer chain version of RGD (CGGNGEPRGDTYRAY) stimulated rat osteoblast matrix mineralisation when attached to quartz through silanisation, while FHRRIKA did not. Interestingly, when an RGD and FHRRIKA combination was used, this improved mineralisation, with higher FHRRIKA ratios identified as the most biologically relevant.

A study from Schuler et al. in 2009 [100] investigated rough titanium samples modified with adsorbed poly-l-lysine-graft-polyethylene glycol doped with either FHRRIKA, KRSR or RGD. This study concluded that while FHRRIKA or KRSR peptides alone promoted cell proliferation compared to controls, they did not reach the efficiency of RGD or RGD and FHRIKKA or KRSR combinations in their ability to upregulate cell proliferation. It is possible this issue may be resolved through the adjustment of peptide concentrations to alter potency of the signal. The Sawyer et al. [38] study first discussed in Section 4.1 also examined combinations of peptides, and concluded that combining RGD with either KRSR or FHRRIKA adsorbed on the surface of HA did not elicit an enhanced response greater than that of the peptides used alone, although as previously discussed, they hypothesised that this may have been due to poor adsorption of the peptides to the surface, and that chemical attachment methods may have improved their results.

Finally, Gentile et al. [128] used a layer-by-layer assembly methodology to coat the surface of PLGA electrospun membranes with a combination of FHRIKKA, KRSR and BMP mimetic peptides, using the peptide’s different osteogenic properties to use in different layers. These layers were designed to follow the bone healing process, with KRSR used in the outer layer to improve cell attachment, BMP-mimetic peptides in the middle to improve differentiation and a final layer of FHRIKKA to improve mineralisation. This material demonstrated excellent in vitro results and was successful in preliminary in vivo study. This novel method highlights the strong potential of combinatory solutions, with consideration to the natural bone healing process.

**Table 7:** The use of peptide combinations to enhance bone regeneration. ↑ = increase, ↓ = decrease, ≈ = no change, - = not reported.

Peptide Sequence + Additional Bioactive Components	Attachment Methodology	Substrate	Cell Type	In Vitro Attachment	In Vitro Spreading	In Vitro Differentiation	In Vitro Mineralisation	In Vivo Efficacy	Publication
**RGD + FHRIKKA**									
GPenGRGDSPCA + (G7 or E7)FHRRIKA	Adsorption	HA	HMSC	↑	≈	-	-	-	[38]
CGGRDGS + CGGFHRRIKA	Silanisation	Titanium	RBMSC	↑	↑	-	-	-	[56]
CGGNGEPRGDTYRAY + CGGFHRRIKA	Silanisation	Quartz	RCO	↑	↑	-	↑	-	[55]
CGGNGEPRGDTYRAY + CGGFHRRIKA	Carbodiimide	Hydrogel	RCO	↑	-	-	-	-	[129]
**RGD + KRSR**									
GPenGRGDSPCA and (G7 or E7)KRSR	Adsorption	HA	HMSC	↑	≈	-	-	-	[38]
RGDS + KRSRGGG	Silanisation	Glass	RCO	↑	-	-	-	-	[87]
KRSRG3	Hydrogel	C_2_S^H^_48_C_2_	MG63	↑	-	-	-	-	[79]
RGD + KRSR	Silanisation	Titanium	SAOS2	↑	↑	-	↑	-	[86]
**RGD + BMP Mimetic**									
GGRGDS + KIPKASSVPTELSAISTLYL	Silanisation	Glass	HMSC	↑	↑	↑	-	-	[54]
GGRGDS + KIPKASSVPTELSAISMLYL	Carbodiimide	PET	MC3T3-E1	↑	-	↑	↑	-	[90]
RTVPKPSSAPTQLNAISTLYF	Carbodiimide	PET	MC3T3-E1	↑	-	↑	↑	-	[90]
RKVGKASSVPTKLSPISILYK	Carbodiimide	PET	MC3T3-E1	↑	-	↑	↑	-	[90]
GRGDSPC + RKIPKASSVPTELSAISMLYL	Carbodiimide	PET	HMSC	↑	↑	-	↑	-	[91]
GGRGDS + KIPKASSVPTELSAISTLYL	Click-chemistry	SAMs	BMSC	↑	↑	-	↑	-	[71]
GGRGDS + KIPKASSVPTELSAISTLYL	Click-chemistry	Hydrogel	RBMSC	↑	-	↑	↑	-	[70]
**FHRIKKA + KRSR + BMP Mimetic**									
CFHRRIKA + CKRSR + NSPVNSKIPKACCVPTELSAI	Carbodiimide + Layer-by-Layer	PLGA/HA	RBMSC	↑	↑	↑	↑	↑	[128]
GFHRRIKA + RKIPKASSVPTELSAISMLYL	Carbodiimide	PET	HMSC	↑	↑	-	↑	-	[91]

These studies demonstrate that while all these peptides have their own positive effects, through combining peptides which have complimentary effects it is possible to further increase their osteogenic properties. Further work, including in vivo study, of these combinations is required to examine how these tailored approaches translate.

## 5. Conclusions

Osteogenic peptides may be utilized to improve the properties of alloplastic bone grafts, with benefits to quality assurance, predictability, cost-effectiveness, and clinical outcomes. These benefits make peptides ideal for use in the bone graft market, with several first-generation products approved and in clinical use. However, these are not yet entirely synthetic or based on covalently bound molecules, and further work is required to reach full clinical maturity while avoiding the adverse events associated with classical orthobiologics. Covalently bound peptides provide the greatest control over dosage, resulting in reduced risk of ectopic effects and greater product stability. Their synthetic nature may also contribute to reduced manufacturing costs while—depending upon the nature of attachment—a medical device classification may be appropriate. In order to fully impact the market, a new generation of wholly synthetic materials must be able to demonstrate consistent clinical results and at least parity with the current ‘gold standard’ of autografting in biological performance. The inherent benefits of a synthetic product over other synthetic graft types, in terms of shelf life, transportation, availability, and a well-defined regulatory pathway, offer a huge market potential once clinical performance is proven.

## Figures and Tables

**Table 1:** The use of RGD peptides to enhance bone regeneration. ↑ = increase, ↓ = decrease, ≈ = no change, - = not reported. Abbreviations: ABM = anorganic bone matrix, BMSC = bone mesenchymal stem cells, HO = human osteoblasts, PET = polyethylene terephthalate, PS = polystyrene, PLGA = poly(lactic-co-glycolic acid), RCO = rat calvaria osteoblast-like, RBMSC = rat BMSCs.

Peptide Sequence + Additional Bioactive Components	Attachment Methodology	Substrate	Cell Type	In Vitro Attachment	In Vitro Spreading	In Vitro Differentiation	In Vitro Mineralisation	In Vivo Efficacy	Publication
GPenGRGDSPCA	Adsorption	HA	HMSC	↑	↓	-	-	-	[37]
RGD	Adsorption	Titanium	SAOS-2	↑	↑	↑	-	-	[47]
GPenGRGDSPCA	Adsorption	HA	HMSC	↑	↓	-	-	-	[38]
RGD	Adsorption	PS	RCO	↑	-	-	-	-	[84]
GRGDSPK	Adsorption	PS	RCO	↑	-	-	-	-	[84]
MAP(RGDSP)	Adsorption	PS	RCO	↑	-	-	-	-	[84]
(GRGDSP)_4_K	Adsorption	ABM	RCO	↑	-	-	-	-	[84]
EEEEEEEPRGDT	Adsorption	HA	MC3T3-E1	↑	↑	-	-	-	[40]
RGDS	Adsorption	Glass	RCO	↑	-	-	-	-	[85]
YRGDSPC	Silanisation	HA	HO	↑	-	-	-	-	[39]
CGGRGDS	Silanisation	Titanium	RBMSC	↑	↑	-	-	-	[56]
RGD	Silanisation	Titanium	SAOS2	↑	↑	-	↑	-	[86]
CGGNGEPRGDTYRAY	Silanisation	Quartz	RCO	↑	↑	-	↑	-	[55]
RGD	Silanisation	HA	MG63	↑	↑	-	-	↑	[52]
GGRGDS	Silanisation	Glass	HMSC	↑	↑	**≈**	-	-	[54]
RGDS	Silanisation	Glass	RCO	≈	-	-	-	-	[87]
RGDS	Carbodiimide	Silk	HO	↑	↑	-	-	-	[88]
GRGDS	Carbodiimide	Silk	SAOS-2	↑	↑	↑	-	-	[89]
RGDSPC	Carbodiimide	PET	MC3T3-E1	↑	-	-	-	-	[90]
GRDSPC	Carbodiimide	PET	HMSC	↑	↑	-	↑	-	[91]
K_16_(GRGDSPC) + BMSCs	Carbodiimide	PLGA	BMSC	-	-	≈	≈	≈	[92]
K_16_(GRGDSPC) + TGF-β + BMSCs	Carbodiimide	PLGA	BMSC	-	-	↑	↑	↑	[92]
GRGDSP	Carbodiimide	Hydrogel	BMSC	↑	≈	↑	-	-	[93]
RGDS	Click-chemistry	SAMs	BMSC	↑	≈	≈	≈	-	[71]
GRGD	Click-chemistry	Hydrogel	RBMSC	↑	-	↑	↑	-	[70]
GRGDS	Hydrogel	PEG	RBMSC	↑	↑	-	-	-	[76]
RGDG	Hydrogel	PEG	RCO	↑	↑	**≈**	↓	-	[29]
GRGDSPGGSGGGSGGGSGGRGDSP	Hydrogel	C_2_S^H^_48_C_2_	MG63	↑	-	-	-	-	[79]
GRGDS + BMP-2	Hydrogel	PEG	HMSC	↑	↑	↑	↑	≈	[78]

**Table 2:** The use of PHSRN peptides to enhance bone regeneration. ↑ = increase, ↓ = decrease, ≈ = no change, - = not reported.

Peptide Sequence + Additional Bioactive Components	Attachment Methodology	Substrate	Cell Type	In Vitro Attachment	In Vitro Spreading	In Vitro Differentiation	In Vitro Mineralisation	In Vivo Efficacy	Publication
PHSRN	Adsorption	Titanium	SAOS-2	≈	≈	≈	-	-	[47]
CGGPHRSN	Silanisation	Titanium	RBMSC	↑	↑	-	-	-	[56]
RGD + PHSRN	Adsorption	Titanium	SAOS-2	↑	↑	≈	-	-	[47]
RGD + PHSRN	Adsorption	Titanium	SAOS-2	↑	↑	≈	-	-	[47]
GRGD + G_13_ + PHSRN	Adsorption	PS	BMSC	↑	-	↑	-	-	[95]
CGGRDGS + CGGPHSRN	Silanisation	Titanium	RBMSC	↑	≈	-	-	-	[56]
GRGD + G_13_ + PHSRN	Carbodiimide	SAMs	MC3T3-E1	↑	-	-	-	-	[96]
GRGD + G_13_ + PHSRN	Hydrogel	PEG	RCO	↑	↑	↑	↓	-	[29]

**Table 3:** The use of FHRIKKA and KRSR peptides to enhance bone regeneration. ↑ = increase, ↓ = decrease, ≈ = no change, - = not reported. Abbreviations: POSS-PCU = polyhedral oligomeric silsesquioxane poly (carbonate-urea) urethane.

Peptide Sequence + Additional Bioactive Components	Attachment Methodology	Substrate	Cell Type	In Vitro Attachment	In Vitro Spreading	In Vitro Differentiation	In Vitro Mineralisation	In Vivo Efficacy	Publication
(G_7_ or E_7_)FHRRIKA	Adsorption	HA	HMSC	↑	↑	-	-	-	[38]
CGGFHRRIKA	Silanisation	Quartz	RCO	↑	↑	-	**≈**	-	[55]
CGGFHRRIKA	Silanisation	Titanium	RBMSC	↑	↑	-	-	-	[56]
FHRRIKA	Carbodiimide	POSS-PCU	BMSC	↑	↑	↑	-	-	[98]
GFHRRIKA	Carbodiimide	PET	HMSC	↑	↑	-	↑	-	[91]
(G_7_ or E_7_)KRSR	Adsorption	HA	HMSC	↑	↑	-	-	-	[38]
KRSR	Adsorption	PS	RCO	↑	-	-	-	-	[84]
KRSR	Adsorption	PS	RCO	↑	-	-	-	-	[84]
MAP(KRSR)	Adsorption	PS	RCO	↑	-	-	-	-	[84]
KRSR	Adsorption	Glass	RCO	↑	-	-	-	-	[85]
KRSRGGG	Silanisation	Glass	RCO	↑	-	-	-	-	[87]
KRSRGYC	Silanisation	Titanium	HO	↑	-	-	-	-	[41]
KRSR	Silanisation	Titanium	SAOS2	↑	↑	-	↑	-	[86]
KRSR	Carbodiimide	Silk	HO	↑	↑	-	-	-	[88]
KRSR	Carbodiimide	POSS-PCU	BMSC	↑	↑	≈	-	-	[98]
FHRIKKA + KRSR	Carbodiimide	POSS-PCU	BMSC	↑	↑	↑	-	-	[98]

**Table 4:** The use of GFOGER peptides to enhance bone regeneration. ↑ = increase, ↓ = decrease, ≈ = no change, - = not reported. Abbreviations: PCL = polycaprolactone.

Peptide Sequence + Additional Bioactive Components	Attachment Methodology	Substrate	Cell Type	In Vitro Attachment	In Vitro Spreading	In Vitro Differentiation	In Vitro Mineralisation	In Vivo Efficacy	Publication
GPC(GPP)_5_GFOGER(GPP)_5_GPC	Adsorption	HA	HMSC	≈	↓	-	-	-	[37]
GGYGGGPC(GPP)_5_ GFOGER	Adsorption	PCL	HMSC	↑	↑	↑	-	-	[106]
GGYGGGPC(GPP)_5_ GFOGER(GPP)_5_GPC	Adsorption	Titanium	BMSC	↑	↑	↑	↑	↑	[104]
GGYGGGPC(GPP)_5_ GFOGER(GPP)_5_GPC	Adsorption	PCL	BMSC	-	-	-	-	↑	[105]
GGYGGGPC(GPP)_5_ GFOGER(GPP)_5_GPC	Carbodiimide	Hydrogel	BMSC	↑	↑	↑	-	-	[93]
GGYGGGPC(GPP)_5_ GFOGER(GPP)_5_GPC	Hydrogel	PEG	HMSC	-	-	-	-	↑	[78]
GGYGGGPC(GPP)_5_ GFOGER(GPP)_5_GPC + BMP-2	Hydrogel	PEG	HMSC	↑	↑	↑	↑	↑	[78]

**Table 5:** The use of P15 peptides to enhance bone regeneration. ↑ = increase, ↓ = decrease, ≈ = no change, - = not reported.

Peptide Sequence + Additional Bioactive Components	Attachment Methodology	Substrate	Cell Type	In Vitro Attachment	In Vitro Spreading	In Vitro Differentiation	In Vitro Mineralisation	In Vivo Efficacy	Publication
GTPGPQGIAGQRGVV	Adsorption	HA	HMSC	↑	↑	↑	↑	↑	[37]
GTPGPQGIAGQRGVV	Adsorption	HA	HMSC	↑	↑	↑	↑	-	[117]
GTPGPQGIAGQRGVV	Silanisation	Titanium	C3H10T1	↑	↑	↑	↑	-	[53]

**Table 6:** The use of BMP mimetic peptides to enhance bone regeneration. ↑ = increase, ↓ = decrease, ≈ = no change, - = not reported. Abbreviations: αTCP = α tricalcium phosphate, PAA = poly(acrylamide-co-acrylic acid), PLA = polylactic acid, PLLA = poly(L-lactic acid), TBC = true bone ceramic.

Peptide Sequence + Additional Bioactive Components	Attachment Methodology	Substrate	Cell Type	In Vitro Attachment	In Vitro Spreading	In Vitro Differentiation	In Vitro Mineralisation	In Vivo Efficacy	Publication
KIPKASSVPTELSAISTLYLDDD	Adsorption	TBC-Collagen	HO	↑	↑	↑	↑	↑	[50]
KIPKASSVPTELSAISTLYL	Adsorption	αTCP	-	-	-	-	-	↑	[43]
KIPKASSVPTELSAISTLYLDDD	Adsorption	HA/Collagen/PLA	-	-	-	-	-	↑	[122]
KIPKASSVPTELSAISTLYLDDD	Adsorption + encapsulation	HA/Collagen/PLLA	HMSC	↑	↑	↑	-	-	[42]
EEEEEEEKIPKASSVPTELSAISTLYL	Adsorption	PLGA	-	-	-	-	-	↑	[123]
KIPKASSVPTELSAISTLYL	Silanisation	Glass	HMSC	↑	↑	↑	-	-	[54]
KIPKASSVPTELSAISTLYLDDD	Carbodiimide	PLGA	-	-	-	-	-	↑	[120]
KIPKASSVPTELSAISTLYLDDD	Carbodiimide	PLGA	HO	↑	↑	↑	↑	↑	[49]
KIPKASSVPTELSAISTLYLDDD	Carbodiimide	Alginate	C3H10T	↑	-	↑	↑	-	[121]
KIPKASSVPTELSAISTLYLDDD	Carbodiimide	Alginate	-	-	-	-	-	↑	[121]
KIPKASSVPTELSAISTLYLDDD	Carbodiimide	Alginate	-	-	-	-	-	↑	[124]
NSVNSKIPKACCVPTELSAI	Carbodiimide	Alginate	-	-	-	-	-	↑	[125]
KIPKASSVPTELSAISMLYL	Carbodiimide	PAA	HMSC	↑	↑	↑	-	-	[126]
KIPKASSVPTELSAISTLYL	Click-chemistry	SAMs	BMSC	↑	↑	↑	≈	-	[71]
KIPKASSVPTELSAISTLYL	Click-chemistry	Hydrogel	RBMSC	↑	-	↑	↑	-	[70]
KIPKASSVPTELSAISTLYL + SVVYGLR	Click-chemistry	Hydrogel	RBMSC	↑	↑	↑	↑	-	[127]

## Data Availability

Data sharing is not applicable to this article.
